# Comprehensive analysis of the flavor volatiles and quality characteristics of ginseng products via GC × GC-TOF-MS, aroma profiles and multivariate statistics

**DOI:** 10.3389/fnut.2025.1719311

**Published:** 2025-12-01

**Authors:** Jun Li, Xinyi Xu, Xinrui Zhou, Xiaowen Li, Wentao Guo, Zhe Lin, He Lin, Yongge Piao

**Affiliations:** 1 College of Pharmacy, Changchun University of Chinese Medicine, Changchun, China; 2 Technology R&D Center, Jilin Tobacco Industry Co., Ltd., Changchun, China

**Keywords:** ginseng products, GC × GC-TOF-MS, volatile compounds, OAV, flavor characteristics

## Abstract

**Introduction:**

As a popular supplement in the world, ginseng occupies a prominent position in the health food industry. With the expansion of the consumer market, ginseng products have gained wider applications. Previous studies have mainly focused on the cultivation, chemical composition, functions and mechanisms of action of ginseng.

**Methods:**

To explore the differences of flavor quality characteristics of ginseng products, the volatile metabolites of seven products were evaluate from different growth years, growth environments, processing methods, and parts by GC × GC-ToF-MS, sensory evaluation and multivariate statistics.

**Results:**

The six-year red ginseng exhibited the highest diversity of volatile metabolites with 2,264 compounds identified, followed by ginseng flowers and ginseng under forest. Odour activity values showed that compounds such as 2,3-Butanedione, Pyrazine, and 2-methoxy-3-(1-methylethyl)-, enhanced the pleasant and buttery flavors of ginseng products. And ginseng flowers displayed more pronounced fruity and citrus flavor characteristics. Additionally, the differences of growing year, growing environment, processing methods and parts on flavor characteristics were analyzed by multivariate statistics. The flavor compounds in ginseng products with a low growth period mainly contribute to sweetness and fruity flavor. Ginseng products after steaming and boiling have more attractive sweet characteristics. The differences in the growth environment significantly affect flavor characteristics including sweetness, greenness, fruity, floral, bitter, herbal and waxy.

**Discussion:**

This study provided a vital reference for the establishment of a convenient detection and identification system linking ginseng raw materials, flavor characteristics, volatile compounds, and product quality control.

## 1 Introduction

Panax ginseng comprises the entire plant, including flowers, fruits, stems, leaves, and roots. Since planted ginseng is typically harvested at 4–6 years in China, the growth year of commercially available products is usually in this range, except for ginseng under forest (1). As a widely popular tonic globally, ginseng has traditionally been used as a supplement for treating neurological disorders, anemia, mental fatigue, forgetfulness, and chronic fatigue, while also enjoying a prominent position in the health food industry (2). Previous research has primarily focused on cultivation, chemical constituents, function, and mechanisms of ginseng. However, with the expansion of the consumer market, ginseng products have gained wider applications. For instance, ginseng flowers are now being included in skincare and haircare products (3, 4). Ginseng roots and stems are utilized for brewing ginseng tea, while ginseng roots and leaves are commonly used in soups. Fermented ginseng health wines have also emerged (5), along with various health food innovations like ginseng coffee and ginseng cola (6). Recent trends indicate a shift toward developing ginseng-related skincare, haircare products, and functional foods and beverages, highlighting the importance of flavor attributes associated with ginseng raw materials. Due to the variations in sensory flavor characteristics resulting from differences in harvest year, growing environment, sourcing parts, and processing methods, the identification of commercially available ginseng products can be complex. Consumers and product developers currently lack a swift and effective method for distinguishing these products in the market. Thus, examining active flavor compounds presents a novel and unique perspective for addressing this challenge.

Ginseng contains a variety of volatile compounds, which are significant secondary metabolites found in higher plants. Plants synthesize and release these volatile compounds to adapt to and resist external environmental disturbances, thereby enhancing their ability to fend off external threats (7). Furthermore, volatile compounds serve as sources of sensory stimulation for humans. They were initially discovered to impart unique natural aromas and flavor characteristics to various fruits, flowers, and vegetables (8). Based on their characteristics, these compounds have been widely used in the production of flavoring agents and preservatives in numerous artificial products (9). For instance, alcohols often enhance overall sweetness, aldehydes frequently contribute fresh and green notes, ketones typically exhibit floral, fruity, and sweet characteristics, while phenolic compounds are associated with strong, undesirable odors (10). Studies have shown that the cocktail lactic acid bacteria can increase the content of volatile flavor compounds during the processing of mountain-cultivated ginseng (11). The deficiency of nitrogen and potassium also had an impact on the aroma of ginseng roots. Nitrogen deficiency inhibited terpenoids, upregulates sulfur compounds and enhanced irritation. Potassium deficiency enhanced the sweet/woody odor through flavonoid biosynthesis and toluene degradation. Double potassium and nitrogen deficiency reduced esters and terpenes, weakening the floral fragrance (12). Another study explored consumers’ acceptance of different processing techniques for red ginseng extract through sensory analysis, including products with various flavors such as ginseng, sweet, and jujube (13). In addition, the evaluation of the sensory characteristics of the American ginseng milk beverage showed that it was brown with increased bitter and metallic taste. By adding vanilla flavor and sucralose, the bitterness of the product could be reduced (14). Currently, comprehensive two-dimensional gas chromatography coupled with time-of-flight mass spectrometry (GC × GC-TOF-MS) is an advanced technique for detecting volatile compounds that was originally developed for the analysis of volatile organic compounds in food and beverages such as meat (15), alcoholic beverages (16), tea (17), and dairy products (18). However, there is a lack of literature utilizing GC × GC-TOF-MS technology to identify and study volatile metabolites in ginseng products.

This study primarily focuses on comparing differential substances and flavor characteristics, providing a flavor-based perspective to expand the development of ginseng products. From the standpoint of flavor compounds, volatile metabolite detection was conducted using the GC × GC-TOF-MS platform in conjunction with the FlavorDB database. The sensory evaluation and multivariate statistical methods were employed to explore the differences in flavor characteristics and flavor compounds among seven ginseng products, as well as the unique key differential compounds associated with these flavor variations. This research aims to clarify the aromatic profiles of commercially available ginseng products, understand the sources of flavor quality differences among them, and provide a reference for establishing a streamlined detection and identification system linking ginseng raw materials, flavor characteristics, differential flavor compounds, product associations, and quality control.

## 2 Materials and methods

### 2.1 Chemicals and reagents

Ethanol was purchased from Aladdin (Shanghai, China). n-Hexyl-d13 Alcohol was obtained from C/D/N Isotopes INC (Quebec, Canada). n-Alkanes was purchased from SIGMA (United States). N-Hexane was purchased from Yonghua (Shanghai, China).

### 2.2 Ginseng samples

Ginseng samples (Panax ginseng C. A. Mey.) were purchased from the WanliangChangbai Mountain Ginseng Market in Jilin Province, China, including seven types as shown in Table 1.

**TABLE 1:** Seven types of ginseng products details.

Type	Growth years	Processing method	Abbreviation
White ginseng	4 years	Sun-dried	WG-4
Red ginseng	4 years	Steamed	RG-4
White ginseng	6 years	Sun-dried	WG-6
Red ginseng	6 years	Steamed	RG-6
Ginseng under forest	6 years	Sun-dried	GUF
Ginseng stem leaf	4 years		GSL
Ginseng flowers	4 years		GF

### 2.3 Extraction of aromatic compounds in ginseng by head-space solid-phase microextraction

#### 2.3.1 Internal standard solutions preparation

Two stock solutions were prepared for the study: one containing 10 mg/L of n-Hexyl-d13 Alcohol dissolved in 50% ethanol, and another containing 10 mg/L of n-Alkanes prepared in n-Hexane. Both solutions were subsequently stored in a refrigerator at 4 °C to maintain their stability.

#### 2.3.2 Extraction of volatile components

The procedure begins with 1 g sample being taken into a 20 mL headspace vial, followed by the addition of 10 μL of the internal standard (ISTD) solution to each sample. The samples are then incubated at 60 °C for 10 min. Before extracting the sample, the Solid Phase Microextraction (SPME) fiber is placed in the chamber at 270 °C for 10 min. Subsequently, the SPME fiber is transferred to the incubator at 60 °C for an additional 15 min. After this incubation, the SPME fiber is desorbed in the gas chromatography (GC) injector at 250 °C for 5 min. Following the desorption process, the SPME fiber is once again placed in the chamber at 270 °C for another 10 min. Finally, 10 μL of n-Alkanes is transferred into the 20 mL headspace vial, completing the extraction and injection steps.

### 2.4 Determination of flavor substances in ginseng by GC × GC-TOF-MS

The LECO Pegasus BT 4D (LECO, St. Joseph, MI, United States) GC × GC-TOF MS system is comprised of an Agilent 8890A gas chromatograph (Agilent Technologies, Palo Alto, CA, United States), a dual-stage jet modulator, and a split/splitless injection module, with the mass spectrometry system featuring a high-resolution TOF detector. The separation system includes a one-dimensional column: DB-Heavy Wax (30 m × 250 μm × 0.5 μm) (Agilent, United States) and a two-dimensional column: Rxi-5Sil MS (2 m × 150 μm × 0.15 μm) (Restek, United States). High-purity helium is utilized as the carrier gas at a constant flow rate of 1.0 mL/min. For the one-dimensional DB-Heavy Wax column, the initial temperature is set at 50°C, held for 2 min, followed by a ramp to 240°C at a rate of 5°C/min, maintained for an additional 5 min. The temperature program for the two-dimensional Rxi-5Sil MS column is set to be 5°C higher than that of the one-dimensional column, and the modulator temperature is consistently maintained at 15°C above the two-dimensional column temperature, with a modulation period of 8.0 s. The injection port temperature is maintained at 250°C. The LECO Pegasus BT 4D mass detector operates with a transfer line temperature of 250°C, an ion source temperature of 250°C, and a collection rate of 200 spectra per second, utilizing an electron impact source at 70 eV, with a detector voltage of 1960V and a mass spectral scanning range of m/z 35–550 (19, 20). The internal standard method was used to calculate the volatile compound content.

### 2.5 Volatile compound qualification and ROAV calculation

This study employed the relative odor activity value (ROAV) method to evaluate the flavor of the samples. Compounds with an ROAV greater than 1 are considered to significantly influence the odor characteristics, with larger ROAV values indicating a greater contribution of the substance to the overall flavor. The ROAV concentration (μg/g) is calculated using the formula ROAV = (C_*i*_/C_*stan*_) × (T_*stan*_/T_*i*_) × 100, where C_*i*_ (%) and T_*i*_ (μg/g) represent the relative percentage content of each volatile substance and the corresponding sensory threshold, and C_*stan*_ (%) and T_*stan*_ (μg/g) represent the relative percentage content and the corresponding sensory threshold of the volatile substance that contributes the most to the overall odor (21).

### 2.6 Analysis of aroma profiles

The FlavorDB database^1^ takes a data-driven approach to identify key features to provide a systematic view of the aroma. It links the chemical properties of volatile compounds with flavors, providing a comprehensive understanding of the flavor profile. In this study, the sensory flavor characteristics were analyzed using the FlavorDB database to assess and compare the sensory profiles of various substances (21, 22). The igraph was used to construct the network relationship graph between them. This analysis aimed to identify both recognizable taste and odor compounds present in the products, as well as complex mixtures that cannot be distinctly identified on their own.

### 2.7 Data processing and multivariate analysis

The measurement of each sample was repeated in parallel three times, and the experimental data are presented as mean values ± standard deviation. The significance levels of metabolites and flavors among various commercial ginseng products were calculated using SPSS 2.0 software through *t*-tests and one-way analysis of variance (ANOVA). Flavor compound annotations were performed on the raw data using the Chroma TOF software in conjunction with the NIST 2020 database (match scores > 80%) (19, 23). Additionally, multivariate analyses, including Principal Component Analysis (PCA), Partial Least Squares Discriminant Analysis (PLS-DA), and Orthogonal Partial Least Squares Discriminant Analysis (OPLS-DA), were conducted on the resulting dataset.

## 3 Results and discussion

### 3.1 Overview of flavor compounds in ginseng products

**FIGURE 1:**
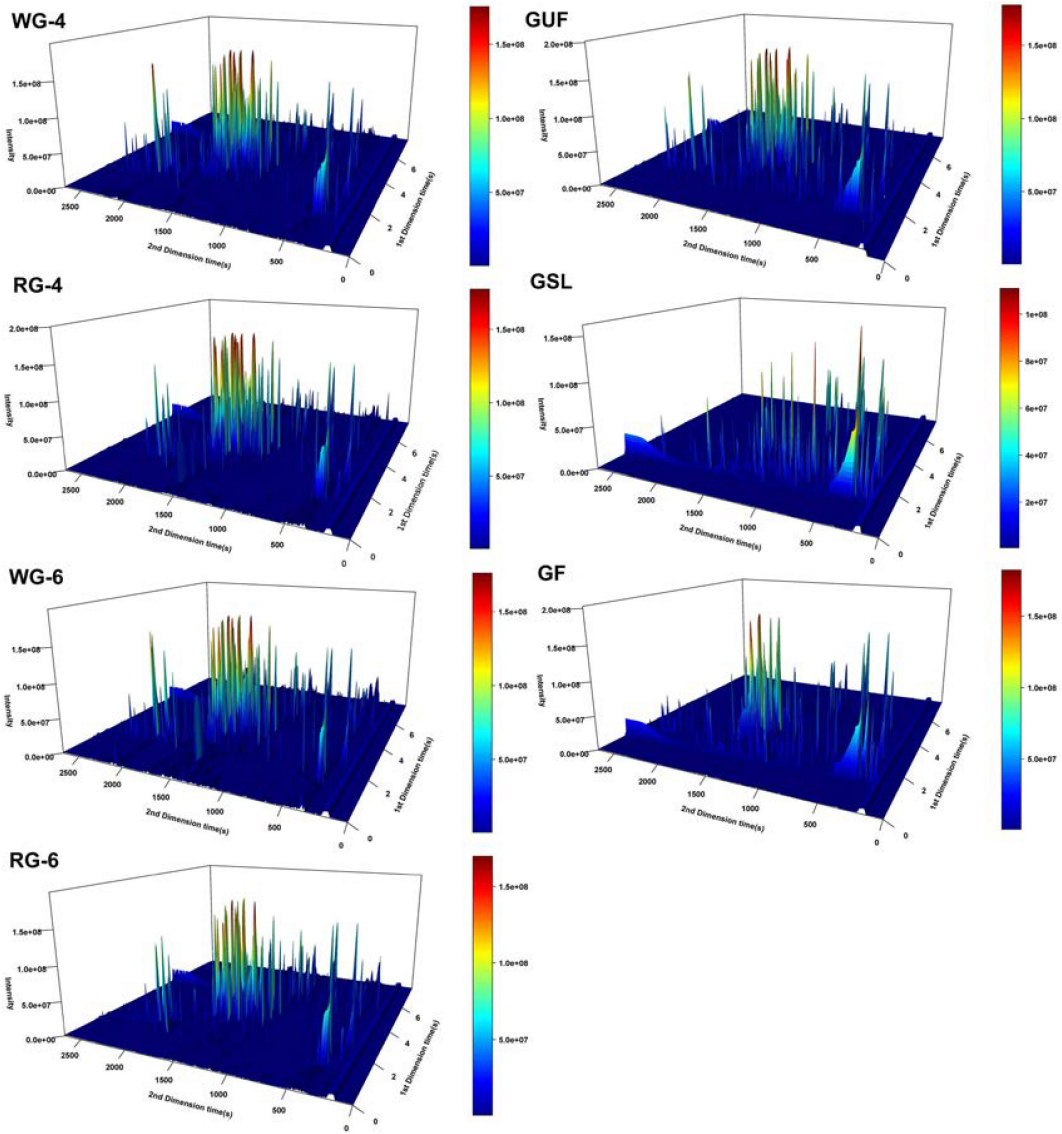
3D total ion chromatogram analysis of volatile compounds in ginseng products using GC × GC-TOF-MS. The x-axis corresponds to one-dimensional retention time (s) and the y-axis represents two-dimensional retention time (s). The color and peak height indicate the intensity of ion response; a deeper red color signifies a higher response intensity.

Ginseng is often described as having a mildly bitter yet sweet flavor accompanied by a distinctive aroma. To investigate the flavor characteristics of these various ginseng products, this study employed GC × GC-TOF-MS technology to analyze the volatile components of the seven selected products. The representative three-dimensional total ion chromatograms of each ginseng product are illustrated in Figure 1. It can be seen that each sample contains a significant number of volatile compounds, with well-defined overall peaks, which indicates that the ginseng product contains a large amount of volatile compounds.

**FIGURE 2:**
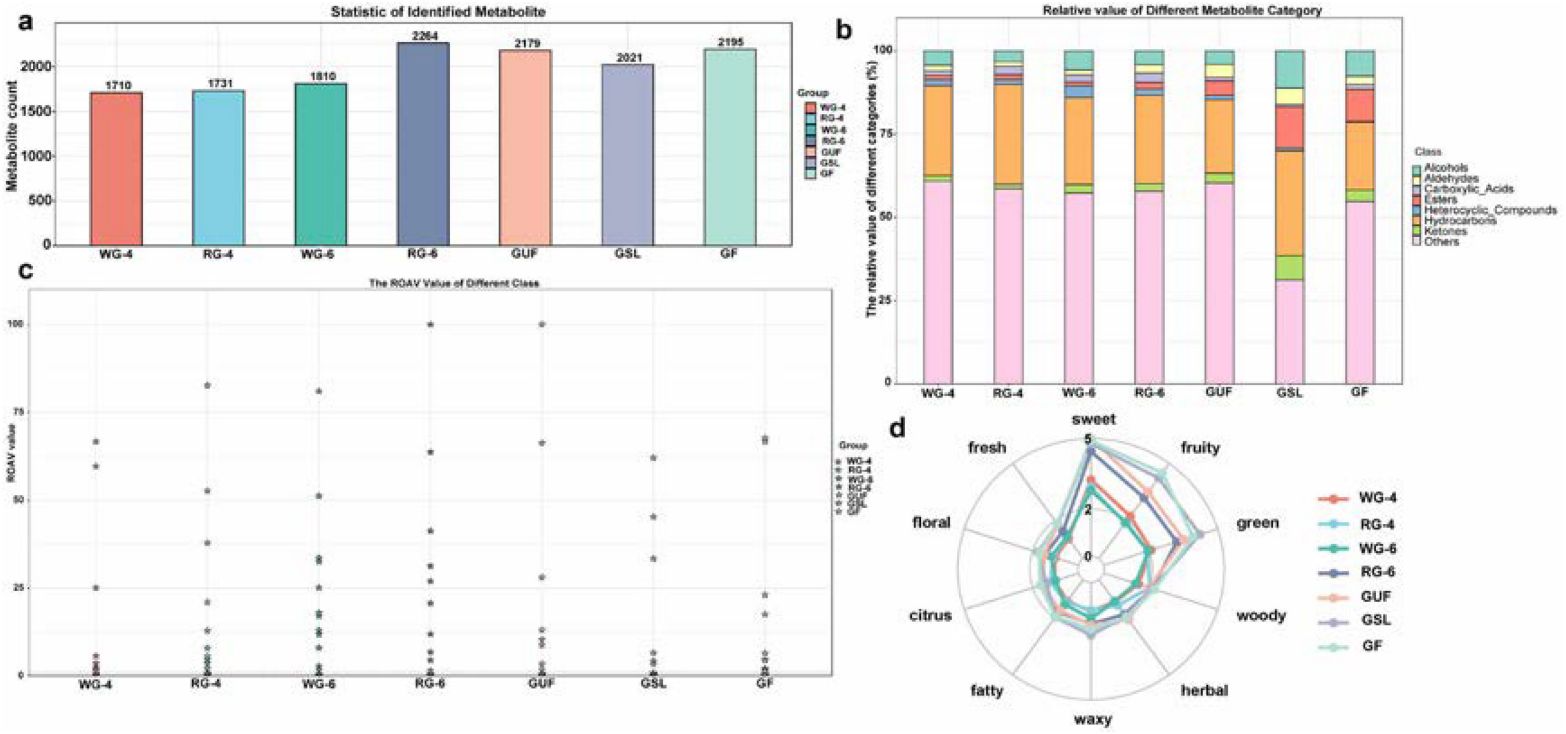
The quantity, relative content, relative odor activity value (ROAV) and sensory preference characteristics of volatile components in ginseng products. **(a)** Number of volatile flavor compounds identified. **(b)** Stacking diagram of the relative content of volatile compounds. **(c)** Scatter plot of ROAV. The x-axis represents the samples of ginseng products, and the y-axis indicates the relative odor activity values (ROAV) of the flavor compounds. Different colors represent various groups. **(d)** Aadar chart of sensory favor characteristics. The outermost circle labels denote the sensory flavor characteristics of the compounds, while the lines represent the frequency ranking of the corresponding flavor compounds (with frequencies classified on a scale from 1 to 5, where the highest frequency is rated as level 5).

As can be seen in Figure 2a, the 4-year cultivated WG-4 and the red ginseng RG-4 showed comparable volatile profiles. However, the 6-year ginseng WG-6 displayed an additional 100 flavor compounds compared to WG-4. In terms of the 6-year group, the RG-6 sample, due to the steaming process, contained the highest number of flavor compounds, totaling 2,264, which is the highest among all seven groups. The GUF, cultivated in a non-artificial environment, exhibited 2,179 volatile flavor compounds, which is higher than those found in WG-4 and WG-6. Additionally, the GSL contained 2,021 flavor compounds, which is 174 fewer than those present in GF.

As shown in Figure 2b and Supplementary Table 1, the volatile flavor compounds detected in ginseng products mainly included hydrocarbons, aldehydes, esters, acids, ketones, alcohols, ethers, phenols and heterocyclic compounds. The relative abundances of these compounds in each sample were also assessed, as represented by stacked bar charts in Figure 2b. The content of flavor compounds in ginseng products increased with the age of the ginseng. Specifically, the relative abundances of alcohols, carboxylic acids, heterocyclic compounds, and ketones rose with year. Alcohols are one of the main volatile compounds in alcoholic products and fermented food, most of which are formed by the metabolism of amino acids or lipid oxidation (24). Unsaturated alcohols with low odor thresholds make a significant contribution to the flavor of food, such as endowing food with wine, fruit and sweet, etc. (25). Heterocyclic compounds are widely present in food and have prominent aroma characteristics, mainly including furan, pyrazine, pyrrole and thiazole, etc. Furans can be produced by Maillard reaction at high temperature, with toasted, nut, almond and other similar aromas (26). Pyrazines generally have a baking aroma, such as toast, baked potatoes, fried peanuts, etc. Ketones are mainly derived from lipid oxidation, amino acid degradation, Maillard reaction and other metabolic reactions, and have fruity and floral fragrance (27). Among products of the same age, red ginseng exhibited a higher quantity of flavor compounds compared to white ginseng, with an increase in the relative abundance of alcohols, heterocyclic compounds, and ketones, while carboxylic acids and esters were less abundant. Carboxylic acids are also important flavor compounds in food, which not only have sour, milky and sweet flavors, but also contribute to sour taste (25). Esters are generally produced by the esterification of organic acids generated from the hydrolysis of alcohols and proteins through esterifying enzymes. It has a low odor threshold, a strong smell and a strong ability to impart fragrance, often generating floral, meaty and other scents (28).

Compared to ginseng cultivated in plantations, GUF grown in a natural mountain environment contained a richer profile of flavor compounds. In this case, the relative abundances of alcohols, carboxylic acids, heterocyclic compounds, and hydrocarbons decreased, while those of aldehydes, esters, and ketones increased. Moreover, ginseng flowers exhibited a higher concentration of flavor compounds than ginseng stems and leaves, with the latter showing elevated levels of alcohols, aldehydes, esters, heterocyclic compounds, hydrocarbons, and ketones when compared to the flowers. These findings suggested that the content of flavor compounds in ginseng increased with age, and the concentration of flavor compounds in red ginseng was higher. Additionally, ginseng flowers were more abundant in flavor substances compared to ginseng stems and leaves. Notably, forest-grown ginseng, despite not being subjected to steaming, still possessed a high content of flavor compounds. This may explain why ginseng grown in a natural mountain environment often has a sweeter taste and superior flavor profile, resulting in better consumer market among commercially available ginseng products. However, it was important to note that these results only reflected the differences in the overall quantities of flavor compounds across the seven types of commercially available ginseng products. Further investigation is required to assess specific compounds and their impacts on the overall flavor characteristics.

### 3.2 Differences of aromatic compounds (ROAV) in ginseng products

To further investigate the contributions and impacts of different flavor compounds on the overall flavor profile of the samples, annotation analysis of the detected flavor compounds were performed using the PubChem database and Classyfire software (29). Additionally, the Relative Odor Activity Value (ROAV) method was employed to evaluate the flavor profiles of the samples. Ultimately, a scatter plot depicting the ROAV values for the aromatic compounds was obtained, as shown in Figure 2c and Table 2.

**TABLE 2:** Odor compounds in ginseng products with ROAVs greater than one.

Volatile compounds	WG-4	RG-4	WG-6	RG-6	GUF	GSL	GF	Odor character
Heptanal	59.67	37.82	25.11	63.65	66.23	61.99	17.55	Citrus, fatty, rancid
2-Nonenal, (E)-	2.19		33.59		13.06	4.24		Fatty, cucumber
2-Octenal, (E)-	25.06	20.91	51.21	26.97	8.76			Nuts, green, fatty
2-Undecanone	1.45	3.60	2.74	4.47			6.46	Orange, fresh, green
a-Pinene	1.62	52.68	11.80	20.70	1.73		4.44	Turpentine, rosiny, pine tree, camphorous, firneedles
Furan, 2-pentyl-	66.67	82.64	12.99	100.00	100.00			Green beans, vegetable
Pyrazine, trimethyl-	5.69	12.87	81.03	41.30				Roasted nuts, cocoa, peanuts
Acetic acid	0.79	5.58	7.99	11.91				Pungent, vinegar
Pyrazine, 2-methoxy-3-(1-methylethyl)-		7.86	17.95	1.46				String-bean, pea, earthy, chocolate, nutty
2,3-Butanedione			32.55		10.31	33.33	66.67	Pleasant, buttery
1-Octen-3-one				31.29	3.40	6.49	23.10	Mushroom-like
Butanoic acid, 3-methyl-						3.33	67.66	Rancid cheese, sweaty, putrid
Acetaldehyde							4.59	Pungent, fruity, suffocating, fresh, green
Propanal, 2-methyl-							1.82	pungent
2-Propenoic acid, ethyl ester							2.07	Sweet, ester, plastic, alcohol, sharp, ammoniacal
Pyrazine, 2-methoxy-3-(1-methylpropyl)-		2.12	1.77				1.48	Nuts, vegetable, green grass, pepper, green beans

To gain a clearer understanding of the sensory flavor characteristics of the ginseng products, the FlavorDB database was utilized to analyze the volatile compounds from different dimensions. By combining their chemical features with molecular flavor profiles, the key flavor attributes in these ginseng products were further identified and radar plots for the sensory flavor characteristics were constructed, as shown in Figure 2d. The results indicated that the primary flavor attributes of the ginseng products are characterized by sweet, fruity, and green notes. The shape of the radar plots revealed a similar trend in the sensory flavor characteristics across the ginseng products. However, there were notable differences in the ratios and intensities of various flavor compounds.

Notably, the forest-grown ginseng (GUF) exhibited stronger sweet and herbal notes, while ginseng flowers, stems and leaves (GSL and GF) demonstrated more pronounced fruity characteristics. In contrast, white ginseng, regardless of age, consistently displayed lower flavor intensity across all attributes compared to other samples. Our preliminary findings highlight both the similarities and distinctions in flavor characteristics among the ginseng products, which are influenced by factors such as the age of the ginseng, processing methods, growing environment, and plant parts used. Previous studies have shown that different drying methods can significantly affect the content of volatile organic compounds in ginseng, which may lead to the loss of odor (30). There are parallels with our results.

In general, the higher the ROAV value, the greater the importance of the compound to the overall aroma. However, not every volatile compound contributes positively to the flavor of ginseng. Different proportions and combinations of flavor compounds can create distinct flavor profiles. In this study, flavor compounds with an ROAV ≥ 1 for each group were filtered and identified as key contributors to the flavor profiles according to the formula in 2.5 (31). As shown in Table 2, the key flavor substances of ginseng products from different cultivation years under the same environmental and processing conditions were analyzed (21, 24). In the WG-4 group, the ROAV values for 2-Nonenal (E), 2-Octenal (E), Heptanal, Butanal (2-methyl), 2-Undecanone, α-Pinene, 2-pentyl Furan, and trimethyl Pyrazine were all greater than 1. In the WG-6 group, the OAV values for 2-Nonenal (E), 2-Octenal (E), Heptanal, Butanal (2-methyl), Acetic acid, 2-Undecanone, 2,3-Butanedione, α-Pinene, 2-methoxy-3-(1-methylpropyl) Pyrazine, 2-methoxy-3-(1-methylethyl) Pyrazine, 2-pentyl Furan, and trimethyl Pyrazine also exceeded 1. The ROAV values of 2-methoxy-3-(1-methylethyl) Pyrazine, trimethyl Pyrazine, Butanal (2-methyl), and 2-Octenal (E) in white ginseng increased with age, enhancing their contributions to flavors such as cocoa and nuts. Meanwhile, 2-Nonenal (E) contributed more significantly to the fatty and cucumber flavors in the ginseng products. Heptanal primarily influenced citrus notes and slightly affected fatty and rancid characteristics without altering the overall flavor profile. However, its contribution to flavor decreased in white ginseng (6 years, WG-6). Additionally, in aged ginseng, the flavor contributions of 2,3-Butanedione and 2-methoxy-3-(1-methylethyl) Pyrazine surged, providing more desirable pleasant and buttery notes while intensifying flavors such as string bean, pea, earthy, chocolate, and nutty. According to the flavor radar map, the short-cultivated WG-4 group performed better than WG-6 group in sweetness, fruity, green and woody properties, but had no significant difference in herbal and citrus aroma. Conversely, the WG-6 group displayed more pronounced floral, fatty, and waxy flavor characteristics than the WG-4 group.

To investigate the changes in flavor characteristics resulting from different processing methods, we conducted an analysis of the WG-4 and RG-4 groups as well as the WG-6 and RG-6 groups. Compared to the WG-4 and WG-6 groups, the contribution of 2-Nonenal (E) to the fatty and cucumber flavors was generally lower in the RG-4 and RG-6 groups. Conversely, α-Pinene played a significant role in contributing to flavor attributes such as turpentine, rosiny, pine tree, camphor, and fir needles in the ginseng products. The ROAV value for 2-pentyl Furan was considerably higher than that of WG-4 and WG-6 groups, primarily influencing the green bean and vegetable notes in the ginseng products. Overall flavor profiles showed that the RG-4 group displayed slightly more pronounced sweet, floral, and citrus notes compared to the WG-4 group, while woody and herbal characteristics were stronger. There were no significant differences in fresh, fruity, waxy, and fatty attributes. In addition, the steam-processed ginseng WG-6 group exhibited significantly higher values for 10 important flavor indicators compared to the WG-6 group, particularly in the sweet, fruity, and green categories. This improvement may be attributed to the flavor contributions of 2-pentyl Furan. It can be preliminarily concluded that steam processing of ginseng with longer cultivation period has superior flavor quality.

Different growing environments often lead to changes and variations in the chemical composition of ginseng. Therefore, the contribution of volatile components to ginseng flavor was explored by comparing WG-4 and WG-6 groups cultivated in farmland with GUF groups grown in forest environments. Flavor compounds with ROAV ≥ 1 in the GUF group were screeded, including 2-Nonenal (E) (Fatty, Cucumber), 2-Octenal (E)-RE (Nuts, Green, Fatty), Heptanal (Citrus, Fatty, Rancid), Butanal (2-methyl) (Cocoa, Almond), 1-Octen-3-one (Mushroom-Like), 2,3-Butanedione (pleasant, buttery), α-Pinene (turpentine, rosiny, pine tree, camphor, fir needles), and 2-pentyl Furan (Green Beans, Vegetable). Comparing the WG-4 and WG-6 groups, it was observed that under-forest the ginseng flavor the contributions of 2-Octenal (E), 2-Undecanone, and trimethyl Pyrazine to the flavor of GUF group reduced, while the contributions of Heptanal and 2-pentyl Furan increased. The unique aromatic compound 1-Octen-3-one contributed mushroom-like flavor characteristics to the GUF group. The overall flavor profiles revealed that the sweet and herbal notes of the ginseng under forest GUF group were the highest among all groups. Compared with WG-4 and WG-6 groups, the GUF group also exhibited more pronounced fruity, green, woody, fresh, and citrus attributes, with minimal differences in floral, fatty, and waxy characteristics.

The contribution of major flavor compounds in stem and leaf (GSL) and flower (GF) of ginseng to the overall taste was compared. In GSL, six compounds exhibited an ROAV ≥ 1, including 2-Nonenal (E) (Fatty, Cucumber), Heptanal (Citrus, Fatty, Rancid), Butanal, 2-methyl- (Cocoa, Almond), 1-Octen-3-one (Mushroom-like), 2,3-Butanedione (pleasant, buttery), and Butanoic acid, 3-methyl- (Rancid Cheese, Sweaty, Putrid). In contrast, GF contained up to ten odor-active compounds with an ROAV ≥ 1, including Acetaldehyde (pungent, fruity, suffocating, fresh, green), Propanal, 2-methyl- (pungent), Heptanal (Citrus, Fatty, Rancid), 2-Propenoic acid, ethyl ester (sweet, ester, plastic, alcohol, sharp, ammoniacal), 1-Octen-3-one (Mushroom-like), 2-Undecanone (Orange, Fresh, Green), 2,3-Butanedione (pleasant, buttery), a-Pinene (turpentine, rosiny, pine tree, camphorous, fir needles), Butanoic acid, 3-methyl- (Rancid Cheese, Sweaty, Putrid), and Pyrazine, 2-methoxy-3-(1-methylpropyl)-. The OAV value of Butanal, 2-methyl- in the stem and leaves of ginseng was significantly higher, contributing cocoa and almond notes to ginseng products. Its flavor contribution in GF was almost negligible. The ROAV of 2, 3-Butanedione, which provided pleasant buttery flavors, was lower in GSL compared to GF. Additionally, it was observed that both 2-Nonenal (E) and Heptanal had higher ROAVs in GSL than in GF. These two flavor compounds significantly influenced the fatty aspects of ginseng. However, the overall difference in the fatty profile between GSL and GF was not pronounced. In ginseng flowers, the ROAV of Butanoic acid, 3-methyl- exceeded that of GSL. This compound primarily contributed unpleasant flavors such as rancid cheese and putrid notes, along with sweaty characteristics, but its overall impact on the flavor profile was limited. The results of sensory flavor characteristics analysis showed revealed that the sensory flavors in GSL and GF were similar in sweet, woody, herbal, fatty, floral, and fresh profiles, while GF exhibited more pronounced fruity and citrus flavor characteristics, with lower levels of green and waxy notes compared to GSL.

### 3.3 Differential flavor compounds in ginseng products and their correlation with flavor

The ginseng products were analyzed using Principal Component Analysis (PCA), which clearly indicated significant differences in the positional distances of the seven sample groups within the model. The R2X values for all samples exceeded 0.5, suggesting notable variations in volatile organic compounds, and indicating that the data is generally reliable with good interpretability. To distinguish the characteristic flavor compounds among the seven commercial ginseng products, the Partial Least Squares Discriminant Analysis (PLS-DA) model was employed (32). This approach simplifies complex data into visualized multidimensional representations, effectively differentiating between the samples. According to the PLS-DA score, apart from the comparison between WG-4 and RG-4, which had an R2X of only 0.372, all other pair wise comparisons yielded R2X values were greater than 0.5, and Q2values were also exceeding 0.5. It indicated substantial inter-group differences among the various commercial ginseng products, and demonstrated the model’s high predictive capability, with notably accurate fitting for all groups except WG-4 and RG-4. Orthogonal Partial Least Squares Discriminant Analysis (OPLS-DA) was utilized, with higher Variable Importance in Projection (VIP) values corresponding to a greater contribution to sample differentiation within the model (33). Therefore, differential compounds with VIP > 1.0 and *p*< 0.05 were selected, which assisted in identifying statistically significant inter-group differences. By integrating this data with the FlavorDB database, the unique sensory flavor characteristics of the flavor compounds were filtered and consolidate. Our analysis focused on the sensory attributes of ginseng, including sweet, bitter, green, herbal, fruity, waxy, and woody flavors, constructing a network graph to illustrate the relationships between sensory flavor characteristics and flavor compounds.

#### 3.3.1 Flavor differences in growing years

The PLS-DA scores for the WG-4 and WG-6 groups yielded R2X = 0.595 and Q2 = 0.755 (Supplementary Figures 1a–e), indicating good model fit and repeatability, with minimal intra-group variation and clear inter-group differences.

**FIGURE 3:**
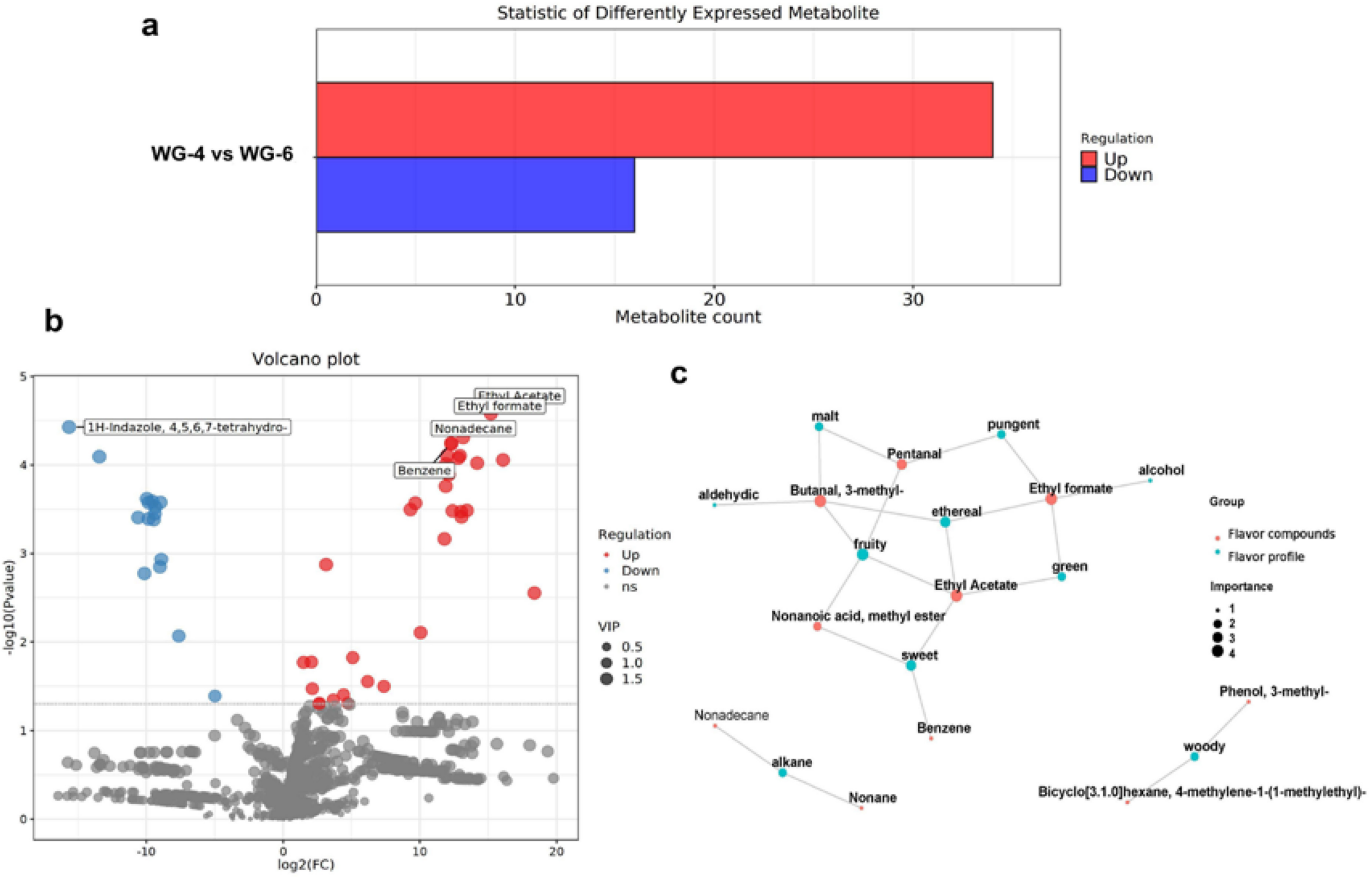
The flavor differences in growing years. **(a)** The number of differential compounds. **(b)** Differential volcano plot for WG-4-WG-6. **(c)** Network diagram illustrating the relationship between sensory flavor characteristics and flavor compounds for WG-4-WG-6.

The results revealed the variation of different compounds in different growing years. By setting the selection criteria (*p* < 0.05, VIP > 1), a total of 50 differential compounds were identified, of which 34 were significantly up-regulated and 16 down-regulated (Figure 3a; Supplementary Figures 1f–h). Compared with WG-6 group, WG-4 group contained significantly higher levels of Butanal,3- methyl-, Ethyl Acetate, Ethyl formate, Cyclohexane, 1,4-dimethyl-2-octadecyl- and 7-epi-α-Eudesmol. And the levels of 3-Nonenoic acid, ethyl ester, Linoleic acid ethyl ester, 1H-Indazole, 4,5,6,7- tetrahydro-, Hexanoic acid, 4-pentenyl ester and Pacifigorgiol were lower (Figure 3b). Subsequently, the relationship between the selected significantly different compounds and their sensory flavor characteristics was analyzed using the FlavorDB database, and eight flavor differentiating compounds were identified (Figure 3c). Notably, Pentanal was found to be present in greater amounts in lower-year ginseng products, contributing unpleasant flavors such as sickening, rancid, decayed, and fermented notes (26). With cultivation years, the relative content of Butanal,3-methyl- decreased, while this compound typically imparts pleasant floral, fruity, and peach-like aromas. Similarly, Ethyl Acetate also decreased with age, primarily enhancing the fruity and sweet flavor characteristics of ginseng. Benzene was reduced with cultivation years, leading to diminished aromatic, sweet, and empyreumatic qualities in ginseng products. Ethyl formate predominantly influenced the aromatic, ethereal, green, rose, pungent, bitter, cognac, and alcohol flavor profiles of ginseng (27). Nonane contributed gasoline and alkane-like flavor notes. Moreover, both Pentana and Ethyl Acetate decreased with longer cultivation times, thereby diminishing the etherous qualities of ginseng products. Among the notable compounds that increased with aging and affected flavor differentiation is Phenol, 3- methyl-, which imparts smoky and petroleum-like characteristics to ginseng products.

Overall, the 4-year white ginseng demonstrated superior sweet, fruity, and green attributes compared to the 6-year white ginseng, showcasing stronger etherouscharacteristics. The constructed flavor compound-flavor characteristic network graph (Figure 3c) revealed that the differences in flavor compounds due to cultivation year primarily influence the overall flavor profile of ginseng products, with a predominant focus on fruity and ethereal notes. Secondary influences include sweet, green, pungent, malt, woody and alkane characteristics. Five key differential compounds such as Butanal, 3- methyl-, EthylAcetate, Ethylformate, Pentanal and Nonanoic acid, methyl ester had significantly impact on flavor. Although individual variations of these compounds may negatively affect the desirability of ginseng products for consumers, collectively, there is a positive contribution to more pleasant sweet and fruiting taste sensations in lower-year ginseng products, enhancing the overall sensory experience.

#### 3.3.2 Flavor differences in processing methods

To investigate the flavor differences in ginseng products resulting from various processing methods (sun-dried and steam-processed treatments), a comparative analysis of the differential compounds between the WG-6-RG-6 groups were conducted. The PLS-DA scores for the WG-6-RG-6 (Supplementary Figures 2a–e) group yielded R2X = 0.625 and Q2 = 0.876. The model demonstrated strong fitting accuracy and good repeatability, with normal distribution of samples within each group.

**FIGURE 4:**
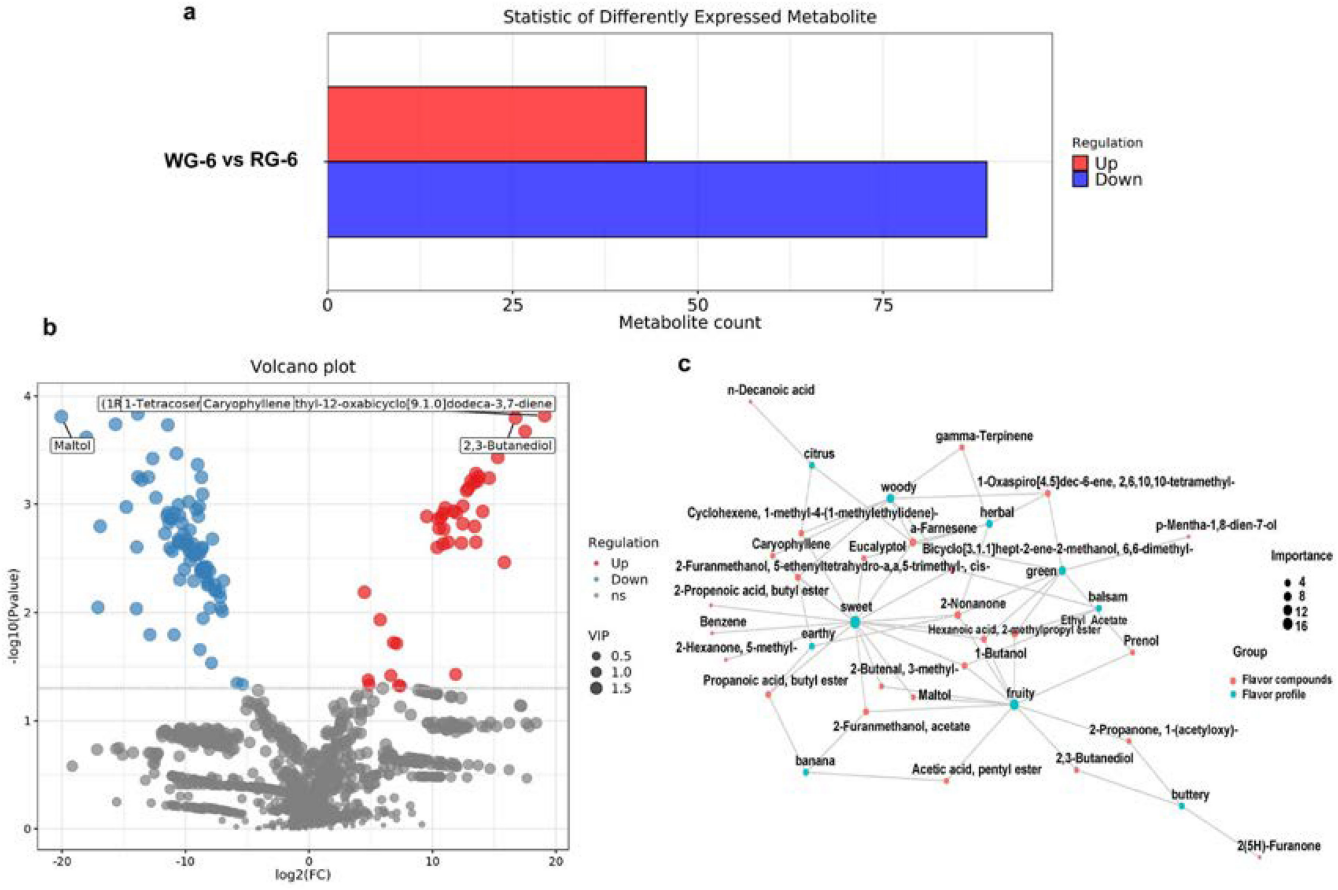
The flavor differences in processing methods. **(a)** The number of differential compounds. **(b)** Differential volcano plot for WG-6-RG-6. **(c)** Network diagram illustrating the relationship between sensory flavor characteristics and flavor compounds for WG-6-RG-6.

Using the selection criteria for differential substances (*p* < 0.05, VIP > 1), it was found that a total of 1,415 flavor compounds exhibited changes between the WG-6 and RG-6 groups, of which 132 differential substances included 43 up-regulated and 89 down-regulated (Figure 4a; Supplementary Figures 2f–h). Among them, 2(5H) -furanone had a high contribution to flavor in the comparisons. The compound has shown bioactivity against various microorganisms and viruses, and potential applications in other medical treatments (34). The relative content of 2(5H)-Furanone was higher in the steamed red ginseng samples, influencing the buttery flavor of ginseng. Cyclohexene, 1-methyl-4-(1-methylethylidene)- also showed increased relative abundance in the red ginseng group, affecting sweetness, woodiness, and citrus notes, thereby providing a more pleasant aroma to the ginseng products (Figure 4b). In steam-processed ginseng products, acetic acid, pentyl ester, and other compounds associated with pungent and bitter characteristics were reduced. Moreover, the creamy flavor of 2,3-butanediol diminished during processing, while maltol, ethyl acetate, 2-furanmethanol acetate, prenol, 3-penten-2-one, 4- methyl-, and 2-nonanoneincreased correspondingly, all of which collectively enhanced fruity and sweet flavors. Furthermore, compared with the WG-6 group, the processing methods may increase gamma-terpinene, 3-methylphenol, 4-ethylphenol, and benzothiazole, resulting in oily, smoky, gasoline-like, leathery, roasted, rubbery, and petroleum-like odors in the RG-6 group.

In terms of the overall effect of different processing methods on flavor, it was mainly concentrated on sweet and fruity characteristics. In the WG-6 and RG-6 groups, significant flavors included green, woody, and herbal flavors, with 2-nonanone, α-farnesene, 1-butanol, ethyl acetate, and hexanoic acid, 2-methylpropyl ester identified as the five principal contributors (Figure 4c).

#### 3.3.3 Flavor differences in growing environment

**FIGURE 5:**
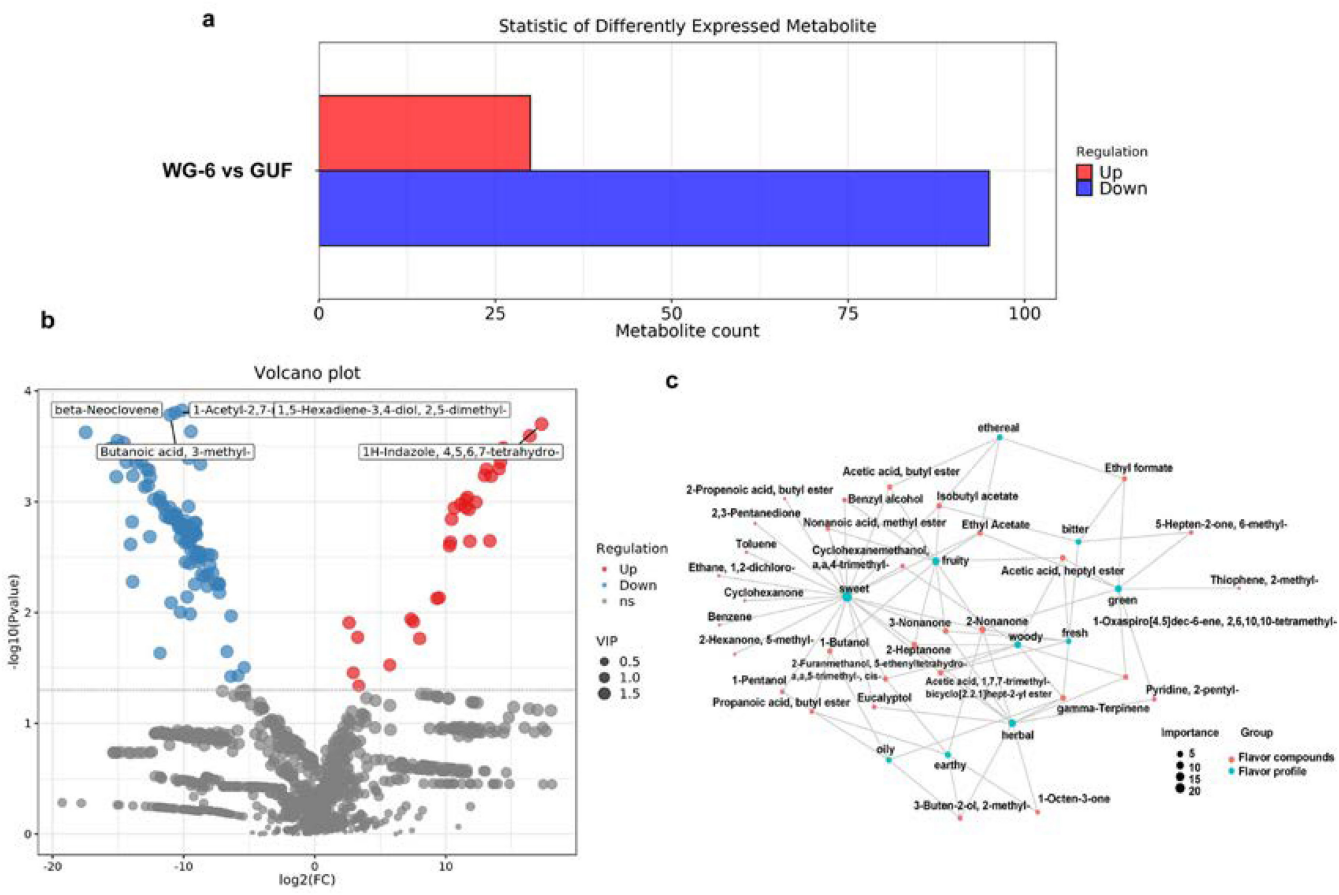
The flavor differences in growing environment. **(a)** The number of differential compounds. **(b)** Differential volcano plot for WG-6-GUF. **(c)** Network diagram illustrating the relationship between sensory flavor characteristics and flavor compounds for WG-6-GUF.

The PLS-DA scores for the WG-6-GUF (Supplementary Figures 3a–e) groups showed R2X = 0.617 and Q2 = 0.917, indicating strong fitting accuracy and good repeatability, with normal distribution of samples within each group. By applying the criteria for differential substance selection (*p* < 0.05, VIP > 1), a total of 125 differential substances were identified across the WG-6-GUF comparisons. Pairwise analysis revealed that 95 substances of WG-6 were down-regulated and 30 were up-regulated compared with GUF (Figure 5a; Supplementary Figures 3f–h).

Utilizing FlavorDB to assess the relationships of these common differential substances with sensory flavor characteristics, four key flavor compounds were identified including 1-Heptanol, 1-Hexanol, 2- Ethyl-, 2-Octenal (E)- and Phenol (Figure 5b). Among these, 1-Hexanol was absent in ginseng cultivated in gardens compared to those grown in forest understories (35). The absence of 1-Hexanol significantly impacted the flavor profiles of ginseng products, enhancing green, rose, oily, fresh, floral, sweet, and citrus notes, and imparting a pleasant fruity aroma. Similarly, 1-Heptanol exhibited low levels regardless of the year of cultivation, particularly in garden vs. forest-grown ginseng. This compound primarily influenced flavor attributes such as green, herbal, violet, leafy, coconut, sweet, peony, chemical, strawberry, and woody, contributing to a scent reminiscent of citrus and oils. Phenol was found at higher concentrations in forest-grown ginseng compared to garden varieties, predominantly affecting medicinal, acid, ink, creosote, and empyreumatic flavors. Additionally, 2-Octenal (E) contributed nutty, fatty, herbal, leaf, green, fresh, banana, cucumber, and waxy notes to ginseng products. Importantly, 2-Octenal (E) is not only a compound influencing ginseng flavor but has also been reported to possess tyrosinase inhibitory activity (36). During mammalian development, tyrosinase is an enzyme critical for melanin production, and excessive accumulation of melanin can lead to various skin disorders (37). Compared to garden ginseng, forest-grown ginseng contained lower levels of 2-Octenal (E), suggesting differences in enzyme inhibition based on cultivation environment. Therefore, when selecting ginseng products on the market, it is necessary to consider both flavor attributes and the need for stronger tyrosinase activity inhibition.

The flavor network map of WG-6-GUF group demonstrated that the difference of growing environment mainly affected the sweet, green, fruity, floral, bitter, herbal and waxy flavor characteristics of ginseng products. The nine most significant substances, 1-Hexanol, 2- Ethyl-, 2-Nonanone, 1-Nonanol, 2-Octenal (E), Decanoic acid ethyl ester, Formic acid heptyl ester, Benzeneacetaldehyde, and 1-Heptanol, played a crucial role in shaping the overall sensory profile of above ginseng products (Figure 5c).

#### 3.3.4 Flavor differences in sourcing parts

**FIGURE 6:**
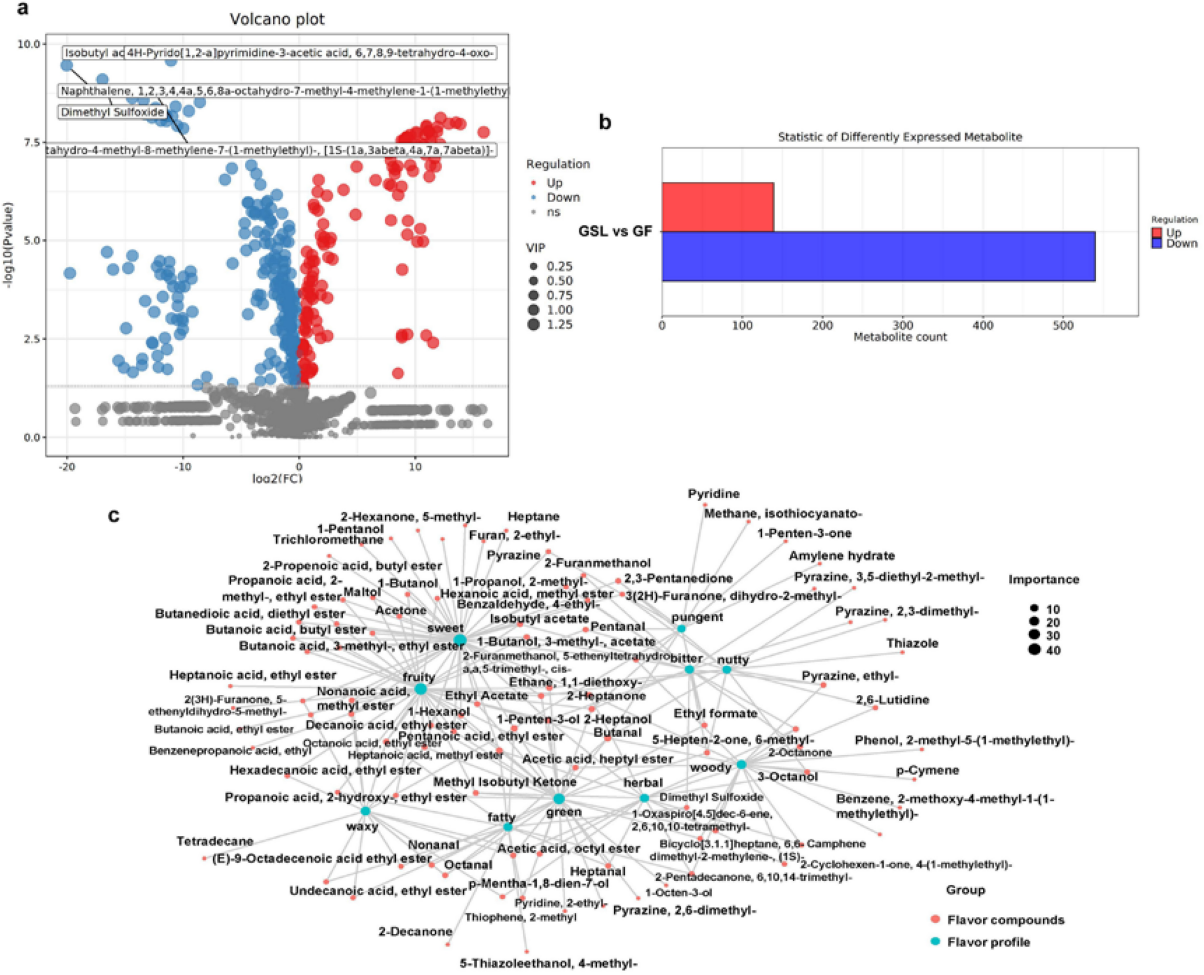
The flavor differences in sourcing parts. **(a)** Differential volcano plot for GSL -GF. **(b)** The number of differential compounds. **(c)** Network diagram illustrating the relationship between sensory flavor characteristics and flavor compounds for GSL -GF.

GSL and GF samples were derived from the stems, leaves, and flowers of ginseng respectively. The PLS-DA scores (Supplementary Figures 3a–e) for these samples were R2X = 0.721 and Q2 = 0.991, indicating a high degree of model fitting accuracy, good repeatability, and normal distribution of samples within groups. To elucidate the Differential substances between the stems and leaves vs. the flowers were screened according to selection criteria (*p* < 0.05, VIP > 1), and their contributions to flavor characteristics were assessed. A total of 679 differential substances were identified when comparing ginseng stems and leaves to ginseng flowers, with 540 showing down-regulation and 139 showing up-regulation in relative abundance. By further refining the selection to |log2*FC*| > 10, 85 significantly differentiating substances were identified (Figures 6a,b; Supplementary Figures 3f–h). Utilizing the FlavorDB database, eight substances that notably impacted the flavor characteristics of ginseng were further found. Among these, five compounds were unique to the ginseng stems and leaves. 2-Propenoic acid primarily contributed sweetness. Acetic acid, butyl ester imparted fruity, ethereal, solvent, pear, and banana notes. Butanal was widely used in the chemical and food industries due to its distinctive aroma, adding pungent, fruity, malty, bready, cocoa, and floral attributes. Ethyl formate enhanced aromatic, ethereal, green, and rose characteristics. Pyrazine, ethyl-, a compound known for its diverse aromas found in potatoes, peanuts, and malt, influenced flavors such as fried sesame seeds, fried peanuts, and fried noodles in our study (38). Accordingly, the ginseng flowers were rich in Isobutyl acetate, contributing fruity, sweet, and apple-like flavors, while Maltol provided sweet, caramel, fruity, and candy flavors. The two substances endowed ginseng flowers with a sweet and fragrant profile, appealing more to consumer preferences. Moreover, Pyrazine, 2,6- dimethyl-, was found at higher concentrations in the ginseng flowers, enhancing the production of flavors such as green pepper fragrant, cocoa, coffee, and roasted nut.

Generally, compared with ginseng flowers, there were a large number of different flavor substances in the ginseng stems and leaves, which significantly affected the overall flavor profiles characterized by sweet, fruity, green, woody, waxy, and pungent notes. And 2-Heptanol, Butanal, Ethyl formate, Ethyl acetate, and Octanoic acid, ethyl ester emerged as five key compounds contributing prominently to the flavor attributes (Figure 6c).

## 4 Conclusion

In this study, the volatile metabolites and flavor characteristics of seven ginseng products were analyzed by GC × GC-TOF-MS, sensory evaluation and multivariate statistics. All groups contained a significant number of volatile compounds, with overall peak appearances being satisfactory. Among the ginseng products, red ginseng (RG-6), which was cultivated for 6 years and subjected to steaming, exhibited the highest number of flavor compounds at 2,264. This was followed by ginseng flowers (GF) with 2,195 compounds, ginseng under forest (GUF) with 2,179 compounds, and ginseng stem leaf (GSL) containing 2,021 flavor compounds. The differences between 4-year white ginseng (WG-4) and steamed red ginseng (RG-4) were minimal, while the higher cultivated year ginseng (WG-6) had 100 more flavor compounds than RG-6. Heptanal was commonly found across all seven ginseng products, contributing citrus, fatty, and rancid flavor characteristics. In the roots and stems/leaves of ginseng, butanal and 2-methyl contributed cocoa and almond flavor notes, with a greater impact observed in the forest-grown ginseng (GUF), whereas these compounds were absent in the ginseng flowers (GF). Key compounds such as (E)-2-octenal, acetic acid, trimethyl pyrazine, and 2-pentyl furan were predominantly present in the roots. Specifically, (E)-2-octenal contributed nutty, green, and fatty flavor aspects to ginseng products WG-4, RG-4, WG-6, RG-6, and GUF, especially to 4-year white ginseng. However, it was not found in ginseng flowers (GF) or stems/leaves (GSL). The steam-processed ginseng exhibited higher levels of 2-undecanone and α-pinene, which significantly enhanced flavor contributions. The former influenced orange, fresh, and green notes, while the latter was a major volatile compound providing turpentine, rosin, pine tree, camphor, and fir needle aromas, potentially explaining the unique flavor and quality of red ginseng. Acetaldehyde and 2-methyl propanal were primarily found in ginseng flowers (GF), with acetaldehyde contributing pungent, fruity, suffocating, fresh, and green flavors, while 2-methyl propanal further intensified the pungent notes in the flowers. Butanoic acid (3-methyl) contributed rancid cheese, sweaty, and putrid flavors, predominantly in ginseng flowers, followed by stems/leaves, while being virtually absent in the roots.

To sum up, flavor compounds in lower-aged ginseng products mainly contribute to sweet and fruity sensations, and have more appealing and sweet flavor characteristics through steaming possess. Differences in growing environment significantly affect flavor traits, including sweet, green, fruity, floral, bitter, herbal, and waxy notes. Ginseng under forest (GUF) exhibited the highest levels of sweet and herbal flavors among all groups. In contrast, cultivated garden ginseng (WG-4 and WG-6) showed more pronounced fruity, green, woody, fresh, and citrusy notes, with similar levels of floral, fatty, and waxy attributes. There were significant variability in flavor compounds between ginseng stems/leaves and flowers, influencing the predominant flavor characteristics of sweet, fruity, green, woody, waxy, and pungent. Ginseng stem leaf (GSL) and ginseng flowers (GF) showed close proximity in sweet, woody, herbal, fatty, floral, and fresh flavors, with GF exhibiting more prominent fruity and citrus characteristics, while green and waxy flavors were slightly lower than those in GSL. These findings enhance our understanding of the relationships between the seven commercially available ginseng products and their corresponding flavor characteristics.

## Data Availability

The original contributions presented in this study are included in the article/Supplementary material, further inquiries can be directed to the corresponding authors.

## Footnotes

1
http://cosylab.iiitd.edu.in/flavordb

## References

[1] Fang X Wang M Zhou X Wang H Wang H Xiao H. Effects of growth years on ginsenoside biosynthesis of wild ginseng and cultivated ginseng. *BMC Genomics.* (2022) 23:325. 10.1186/s12864-022-08570-0 35461216 PMC9035264

[2] Bahrke MS Morgan WR. Evaluation of the ergogenic properties of ginseng: an update. *Sports Med.* (2000) 29:113–33. 10.2165/00007256-200029020-00004 10701714

[3] Kim JH Lee R Hwang SH Choi SH Kim JH Cho IH Ginseng and ginseng byproducts for skincare and skin health. *J Ginseng Res.* (2024) 48:525–34. 10.1016/j.jgr.2024.09.006 39583168 PMC11583465

[4] Nakamura S Sugimoto S Matsuda H Yoshikawa M. Medicinal flowers. XVII. New dammarane-type triterpene glycosides from flower buds of American ginseng, *Panax quinquefolium* L. *Chem Pharm Bull (Tokyo).* (2007) 55:1342–8. 10.1248/cpb.55.1342 17827759

[5] Yu L Xu Z Qu L. Production of lemon-ginseng superfine powder wine (in Chinese). *Liquor-making Sci Technol.* (2018) 10:76–80.

[6] Yue Y Yin J Xie J Wu S Ding H Han L Comparative analysis of volatile compounds in the flower buds of three Panax species using fast gas chromatography electronic nose, headspace-gas chromatography-ion mobility spectrometry, and headspace solid phase microextraction-gas chromatography-mass spectrometry coupled with multivariate statistical analysis. *Molecules.* (2024) 29:602. 10.3390/molecules29030602 38338347 PMC10856343

[7] Dudareva N Klempien A Muhlemann JK Kaplan I. Biosynthesis, function and metabolic engineering of plant volatile organic compounds. *New Phytol.* (2013) 198:16–32. 23383981 10.1111/nph.12145

[8] Wang Y Yang C Li S Yang L Wang Y Zhao J Volatile characteristics of 50 peaches and nectarines evaluated by HP-SPME with GC-MS. *Food Chem.* (2009) 116:356–64.

[9] Li C Li X Liang G Xiang S Han G. Volatile composition changes in lemon during fruit maturation by HS-SPME-GC-MS. *J Sci Food Agric.* (2022) 102:3599–606. 34873698 10.1002/jsfa.11706

[10] Guan S Liu C Yao Z Wan H Ruan M Wang R Detection and analysis of VOCs in cherry tomato based on GC-MS and GC×GC-TOF MS techniques. *Foods.* (2024) 13:1279. 10.3390/foods13081279 38672951 PMC11048788

[11] Lee HY Lee JH Shin EC Cho DY Jung JG Kim MJ Changes in chemical compositions and antioxidant activities from fresh to fermented red mountain-cultivated ginseng. *Molecules.* (2022) 27:4550. 10.3390/molecules27144550 35889423 PMC9322814

[12] Cao W Sun H Shao C Long H Cui Y Sun C Exploring the effects of nitrogen and potassium on the aromatic characteristics of ginseng roots using non-targeted metabolomics based on GC-MS and multivariate analysis. *Foods.* (2025) 14:2981. 10.3390/foods14172981 40941097 PMC12427790

[13] Yoon EK Hong JH Lê S Kim KO. Sensory characteristics and consumer acceptability of red ginseng extracts produced with different processing methods. *J Food Sci.* (2011) 76:S270–9. 10.1111/j.1750-3841.2011.02197.x 22417441

[14] Tárrega A Salvador A Meyer M Feuillère N Ibarra A Roller M Active compounds and distinctive sensory features provided by American ginseng (*Panax quinquefolius* L.) extract in a new functional milk beverage. *J Dairy Sci.* (2012) 95:4246–55. 10.3168/jds.2012-5341 22818438

[15] Liu H Hui T Fang F Li S Wang Z Zhang D. The formation of key aroma compounds in roasted mutton during the traditional charcoal process. *Meat Sci.* (2022) 184:108689. 10.1016/j.meatsci.2021.108689 34653802

[16] Liu Z Yang K He Z Zhao D Zheng J Qian MC. Comparison of two data processing approaches for aroma marker identification in different distilled liquors using comprehensive two-dimensional gas chromatography-time-of-flight mass spectrometry dataset. *J Food Sci.* (2023) 88:2870–81. 10.1111/1750-3841.16624 37282742

[17] Deng X Huang G Tu Q Zhou H Li Y Shi H Evolution analysis of flavor-active compounds during artificial fermentation of Pu-erh tea. *Food Chem.* (2021) 357:129783. 10.1016/j.foodchem.2021.129783 33892356

[18] Li J Fu Y Bao X Li H Zuo J Zhang M Comparison and analysis of tomato flavor compounds using different extraction methods. *J Food Measure Char.* (2020) 14: 465–75.

[19] Li H Geng W Haruna SA Zhou C Wang Y Ouyang Q Identification of characteristic volatiles and metabolomic pathway during pork storage using HS-SPME-GC/MS coupled with multivariate analysis. *Food Chem.* (2022) 373:131431. 10.1016/j.foodchem.2021.131431 34700034

[20] Sun C Wang R Wang T Li Q. Primary evaluation of nine volatile N-nitrosamines in raw red meat from Tianjin, China, by HS-SPME-GC-MS. *Food Chem.* (2020) 310:125945. 10.1016/j.foodchem.2019.125945 31837529

[21] Chen Y Tao X Hu S He R Ju X Wang Z Effects of phytase/ethanol treatment on aroma characteristics of rapeseed protein isolates. *Food Chem.* (2023) 431:137119. 10.1016/j.foodchem.2023.137119 37572486

[22] Garg N Sethupathy A Tuwani R Nk R Dokania S Iyer A FlavorDB: a database of flavor molecules. *Nucleic Acids Res.* (2018) 46:D1210–6. 29059383 10.1093/nar/gkx957 PMC5753196

[23] Cody RB Sparkman OD Moore H. Creating a searchable chromatographic database with the NIST mass spectral search program. *J Am Soc Mass Spectrom.* (2022) 33:740–3. 10.1021/jasms.2c00016 35262364

[24] Zhang Q Zhang F Gong C Tan X Ren Y Yao K Physicochemical, microbial, and aroma characteristics of Chinese pickled red peppers (*Capsicum annuum*) with and without biofilm. *RSC Adv.* (2020) 10:6609–17. 10.1039/d0ra00490a 35496022 PMC9049736

[25] Lin L Fan W Xu Y Zhu D Yang T Li J. Characterization of key odorants in chinese texiang aroma and flavor type Baijiu (chinese liquor) by means of a molecular sensory science approach. *J Agric Food Chem.* (2024) 72:1256–65. 10.1021/acs.jafc.3c07053 38169436

[26] Yan Y Chen S He Y Nie Y Xu Y. Quantitation of pyrazines in Baijiu and during production process by a rapid and sensitive direct injection UPLC-MS/MS approach. *LWT.* (2021) 128:109371.

[27] Wang L Gao Y Wu L Chen S Xu Y. Characterization of key aging aroma compounds in aged Jiangxiangxing Baijiu and their formation influencing factors during the storge process. *J Agric Food Chem.* (2024) 72:1695–707. 10.1021/acs.jafc.3c06929 38194670

[28] Gao W Fan W Xu Y. Characterization of the key odorants in light aroma type Chinese liquor by gas chromatography-olfactometry, quantitative measurements, aroma recombination, and omission studies. *J Agric Food Chem.* (2024) 62:5796–804. 10.1021/jf501214c 24909925

[29] Djoumbou Feunang Y Eisner R Knox C Chepelev L Hastings J Owen G ClassyFire: automated chemical classification with a comprehensive, computable taxonomy. *J Cheminform.* (2016) 8:61. 27867422 10.1186/s13321-016-0174-y PMC5096306

[30] Xiang Y Zou M Ou F Zhu L Xu Y Zhou Q A comparison of the impacts of different drying methods on the volatile organic compounds in ginseng. *Molecules.* (2024) 29:5235. 10.3390/molecules29225235 39598624 PMC11596846

[31] Zhong Q Xing Z Teng F Wu T Pan S Xu X. Evaluation of the aroma and taste contributions of star anise (*I. Verum hook. f.*) in braised duck leg via flavor omics combined with multivariate statistics. *Food Res Int.* (2024) 184:114209. 10.1016/j.foodres.2024.114209 38609210

[32] Boulesteix AL Strimmer K. Partial least squares: a versatile tool for the analysis of high-dimensional genomic data. *Brief Bioinform.* (2007) 8:32–44. 10.1093/bib/bbl016 16772269

[33] Stenlund H Gorzsás A Persson P Sundberg B Trygg J. Orthogonal projections to latent structures discriminant analysis modeling on in situ FT-IR spectral imaging of liver tissue for identifying sources of variability. *Anal Chem.* (2008) 80:6898–906. 10.1021/ac8005318 18714965

[34] Żurawska K Byczek-Wyrostek A Kasprzycka A Walczak K. 3,4-dihalo-5-hydroxy-2(5H)-furanones: highly reactive small molecules. *Molecules.* (2024) 29:5149. 10.3390/molecules29215149 39519788 PMC11547709

[35] Xi BN Zhang JJ Xu X Li C Shu Y Zhang Y Characterization and metabolism pathway of volatile compounds in walnut oil obtained from various ripening stages via HS-GC-IMS and HS-SPME-GC-MS. *Food Chem.* (2024) 435:137547. 10.1016/j.foodchem.2023.137547 37769558

[36] Wu X Wei F Ding F Yang N Niu J Ran Y Phytochemical analysis, antioxidant, antimicrobial, and anti-enzymatic properties of *Alpinia coriandriodora* (sweet ginger) rhizome. *Front Plant Sci.* (2023) 14:1284931. 10.3389/fpls.2023.1284931 37936928 PMC10626549

[37] Sagun S Collins E Martin C Nolan EJ Horzempa J. Alarm odor compounds of the brown marmorated stink bug exhibit antibacterial activity. *J Pharmacogn Nat Prod.* (2016) 2:119. 10.4172/2472-0992.1000119 27656692 PMC5027987

[38] Feng X Hua Y Li X Zhang C Kong X Chen Y. (E)-2-heptenal in soymilk: a nonenzymatic formation route and the impact on the flavor profile. *J Agric Food Chem.* (2020) 68:14961–9. 10.1021/acs.jafc.0c06192 33249836

