# Supplementary material for: Comprehensive analysis of the flavor volatiles and quality characteristics of ginseng products via GC × GC-TOF-MS, aroma profiles and multivariate statistics
Source: Front Nutr. 2025 Dec 1;12:1719311. doi: 10.3389/fnut.2025.1719311 (PMC12702956; doi:10.3389/fnut.2025.1719311)
Supplement: Supplementary file 1 [file Data_Sheet_1.pdf]

Table S1 The relative content of flavor substances

Types	Relative content (%)							
	**Alcohols**	**Aldehydes**	**Carboxylic _Acids**	**Esters**	**Heterocyclic Compounds**	**Hydrocarbons**	**Ketones**	**Others**
WG-4	4.307	1.750	1.286	1.305	1.797	26.987	1.728	60.842
RG-4	3.273	1.368	2.450	1.475	1.470	30.014	1.299	58.651
WG-6	5.703	1.514	2.189	1.131	3.496	26.113	2.568	57.287
RG-6	4.154	2.429	2.927	1.912	1.898	26.546	2.320	57.814
GUF	3.989	4.018	1.025	4.323	1.339	22.039	2.933	60.336
GSL	11.114	4.943	0.714	12.431	0.869	31.408	7.293	31.228
GF	7.641	2.436	1.509	9.612	0.188	20.367	3.498	54.749

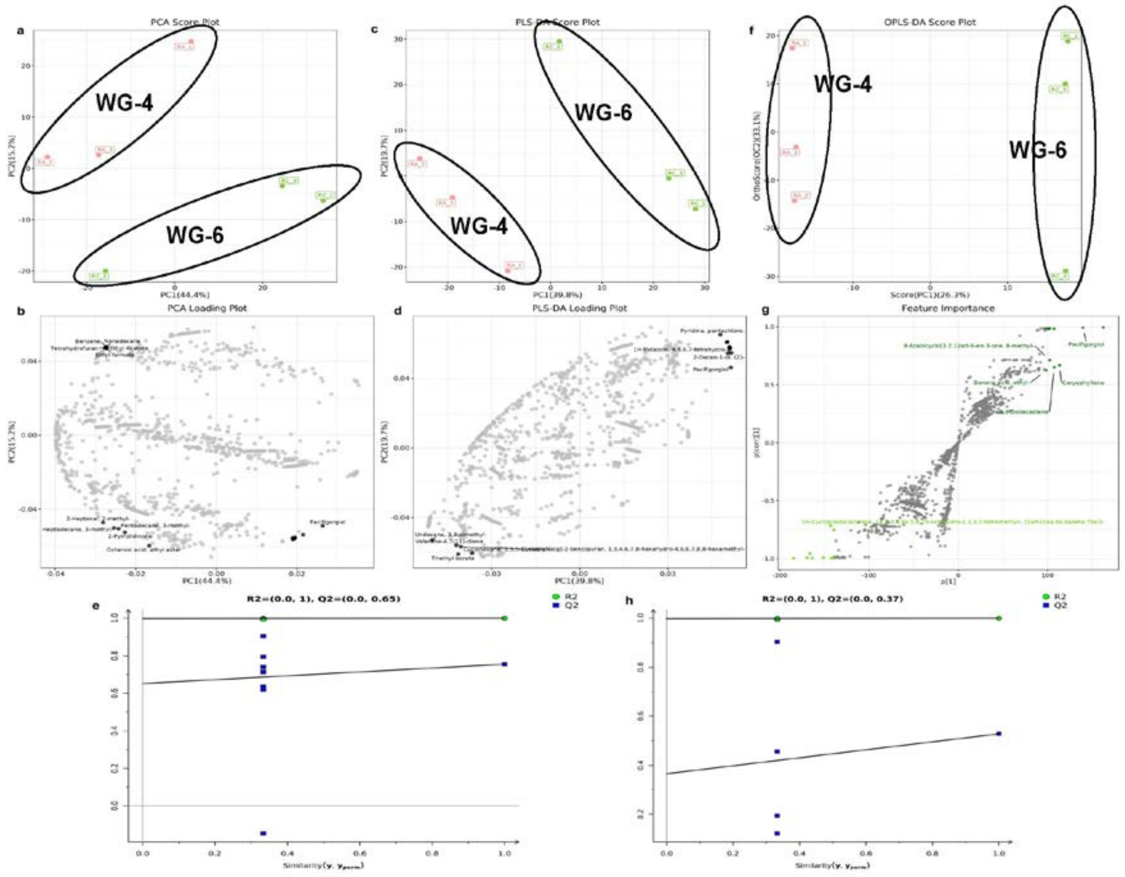

Fig. S1 (a) PCA score plot of WG-4-WG-6. (b) PCA loading plot of WG-4-WG-6. (c) PLS-DA score plot of WG-4-WG-6. (d) PLS-DA loading plot of WG-4-WG-6. (e) PLS-DA permutation test of WG-4-WG-6. (f) OPLS-DA score plot of WG-4-WG-6. (g) OPLS-DA S-plot of WG-4-WG-6. (h) OPLS-DA permutation test of WG-4-WG-6.

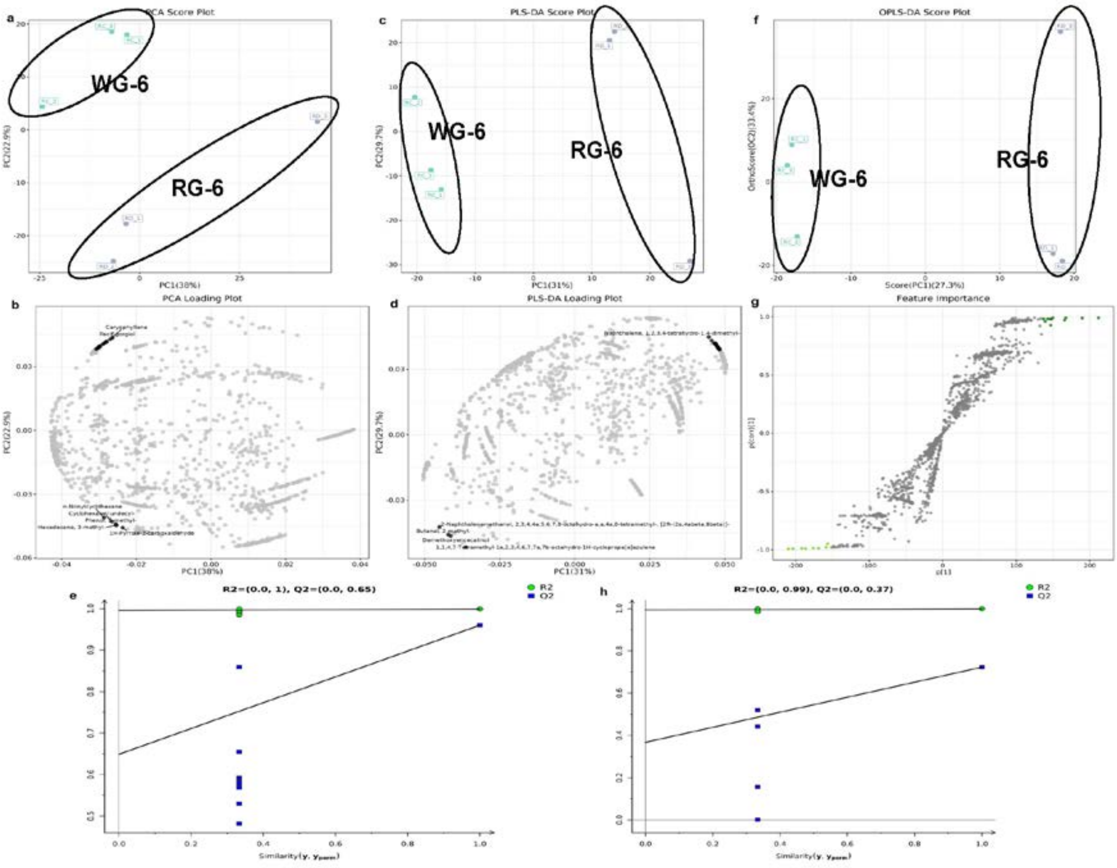

Fig. S2 (a) PCA score plot of WG-6-RG-6. (b) PCA loading plot of WG-6-RG-6. (c) PLS-DA score plot of WG-6-RG-6. (d) PLS-DA loading plot of WG-6-RG-6. (e) PLS-DA permutation test of WG-6-RG-6. (f) OPLS-DA score plot of WG-6-RG-6. (g) OPLS-DA S-plot of WG-6-RG-6. (h) OPLS-DA permutation test of WG-6-RG-6.

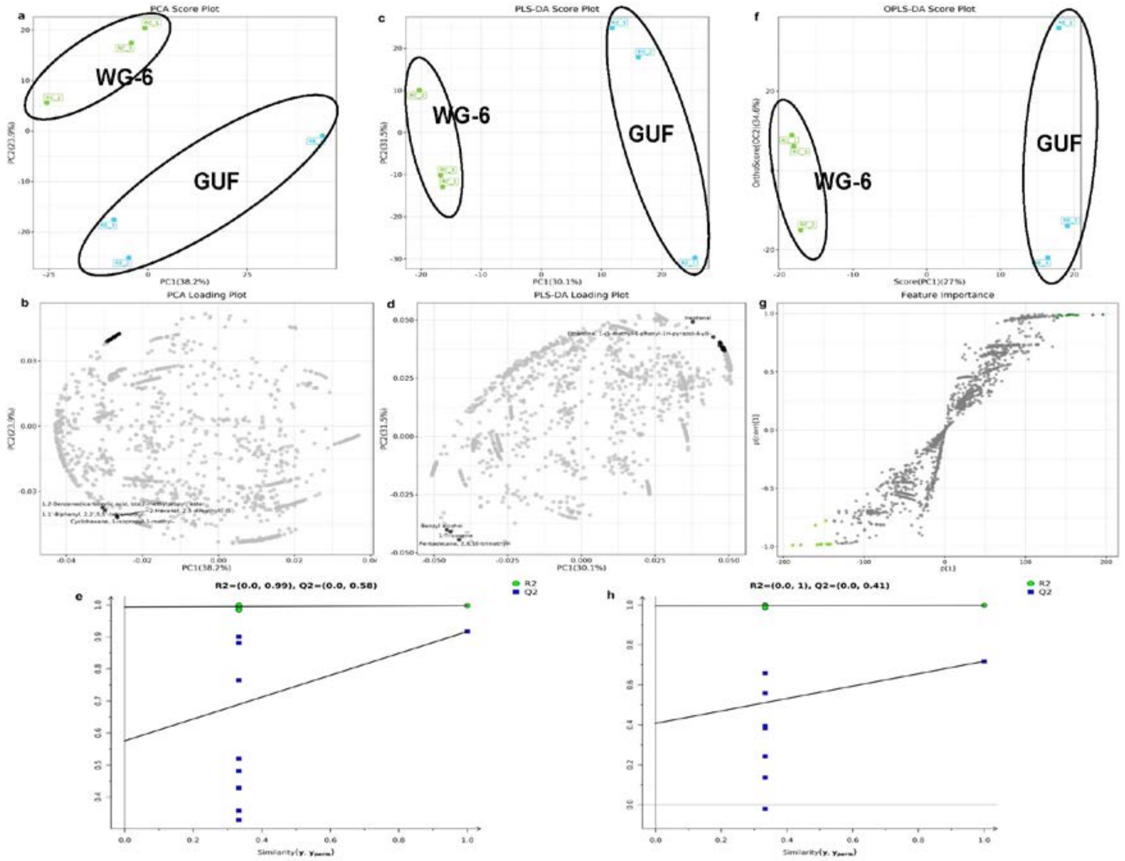

Fig. S3 (a) PCA score plot of WG-6-GUF. (b) PCA loading plot of WG-6-GUF. (c) PLS-DA score plot of WG-6-GUF. (d) PLS-DA loading plot of WG-6-GUF. (e) PLS-DA permutation test of WG-6-GUF. (f) OPLS-DA score plot of WG-6-GUF. (g) OPLS-DA S-plot of W G-6-GUF. (h) OPLS-DA permutation test of WG-6-GUF.

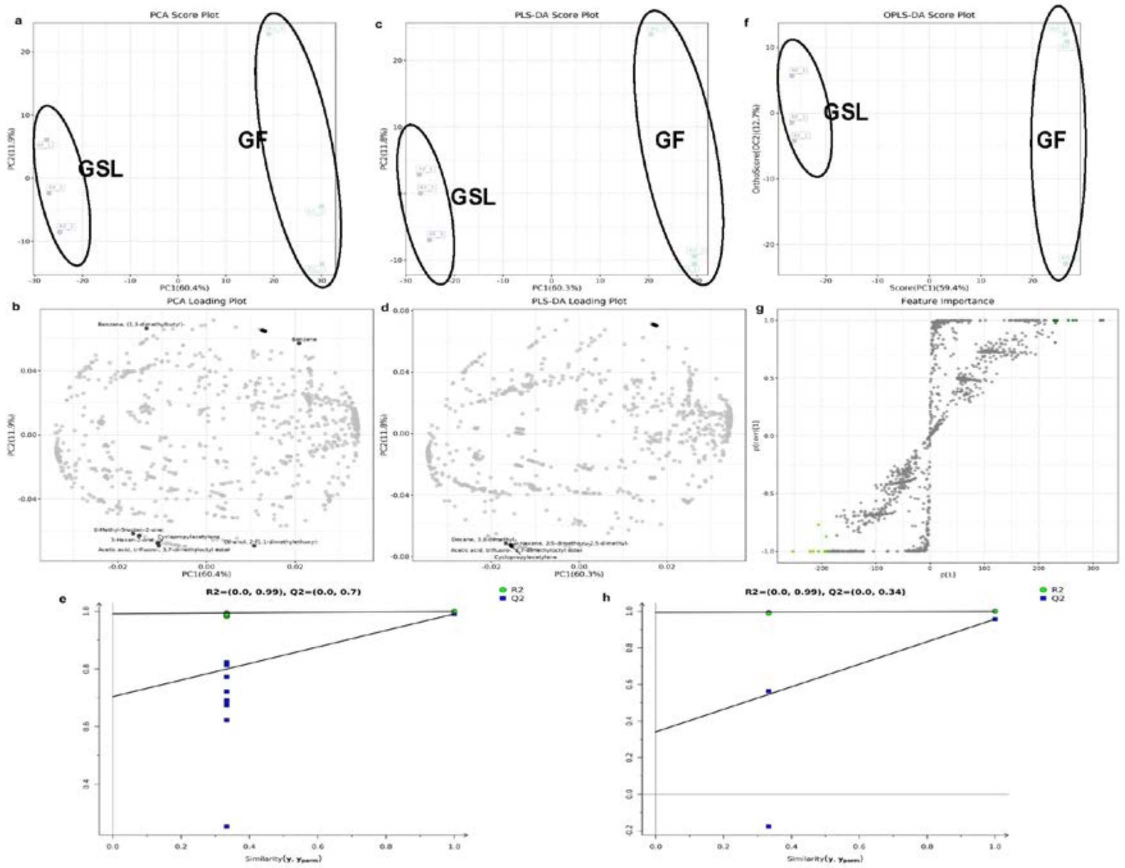

Fig. S4 (a) PCA score plot of GSL-GF. (b) PCA loading plot of GSL-GF. (c) PLS-DA score plot of GSL-GF. (d) PLS-DA loading plot of GSL-GF. (e) PLS-DA permutation test of GSL-GF. (f) OPLS-DA score plot of GSL-GF. (g) OPLS-DA loading plot of GSL-GF. (h) OPLS-DA permutation test of GSL-GF.
